# New York Odontological Society

**Published:** 1887-11

**Authors:** 


### NEW YORK ODONTOLOGICAL SOCIETY.

REPORTED FOR THE INDEPENDENT PRACTITIONER.

The first meeting of this society for the present season was held at the New York Academy of Medicine, Tuesday evening, Oct. 3d., Dr. J. Morgan Howe occupying the chair.

Dr. W. H. Dwinelle exhibited a plaster model, representing what he termed a "giant mouth," being in fact casts of superior and inferior dental arches from the mouth of his colored servant. The arches were uncommonly large, showing in both thirty-four well-shaped teeth. Dr. Dwinelle also exhibited two plaster casts of a female face, taken respectively before and after regulating had been done for correcting protrusion of the upper teeth. Casts representing the teeth of the same case were also exhibited, the first showing a V-shaped, contracted superior arch, with incisors projecting so far forward as to disfigure or raise the lip. The second cast portrayed the corrected condition, the result of spreading the arch on either side and bringing down the front teeth, making a well-formed mouth and face. The doctor also passed for inspection an incisor tooth, once badly broken, but the contour beautifully restored, in which operation he used crystal gold, and to anchor the filling had inserted within the cavity a gold screw.

Dr. W. Jarvie thought Dr. Dwinelle's restoration could have been better accomplished with a porcelain crown.

Dr. Davenport presented a plaster cast, representing the mouth of a young man who had his molar teeth extracted by a dentist in the country simply because they had "ached." This loss of the molars had caused such a degree of pressure on the front teeth as to chip their cutting edges, and to force them forward. Dr. Davenport had been treating the same case for pyorrhœa alveolaris, which also occurred. He introduced a gold plate, covering the roof of the mouth and held in place by atmospheric pressure and clasps. This plate had wings or flanges to cover the bicuspids and a single remaining molar, thus taking much pressure from the front teeth during the process of mastication. He thought the case might be benefited by a rubber plate covering the same teeth for night wear.

The President announced the subject for consideration: "The Combination of Metals as a Filling Material."

A paper from Dr. S. B. Palmer, of Syracuse, was read by the Secretary.

From careful experiment and trial of combination fillings, Dr. Palmer was able to recommend them highly as tooth-saving agents, and particularly valuable did he consider the combination of tin and gold. He described in detail his method of preparing and manipulating it. He places a sheet of tin foil on a sheet of gold foil, and in cutting through them the shears joins together the edges of both, so that in this way united the strips can easily be rolled into cylinders. He spoke of the great advantage this filling had over amalgam in cases where the edges of the cavity walls were thin and friable, and also in "submarine" operations. But where the tooth-structure is firm and compact, he uses gold alone, considering it in favorable cases the best filling material.

Dr. C. T. Stockwell, of Springfield, Mass., sent a paper, which was also read by the Secretary and listened to by the society with great interest. The doctor considered the value of combination fillings over single metals as very great, especially when considering the saving of tooth-structure. He believes that in many instances the combination fillings are more comfortable to the wearer than fillings of one metal, and are often of greater durability. He has used amalgam and gold together, but tin and gold possess this advantage—they can be inserted at one sitting. In badly broken-down teeth he has used amalgam for partly filling the cavities, and after sufficiently hard, has trimmed and cut away a portion of it, then completed the filling with gold, thus preventing the amalgam from showing wholly or in part.

He considers gold excellent for strong teeth, but prefers the combination filling for weak ones. The latter is also valuable for the prevention of trouble from thermal action. He had used such fillings in a great number of instances where pulps were nearly exposed, with no after-trouble from pulp-irritation and loss of vitality, as was often the case where gold had been used. He believes that such chemical conditions exist, that even should a pulp die under a combination filling, abscess would be prevented. He has no doubt that the two metals form an electric current in which micro-organisms cannot exist, and which thus prevents fermentation. This electric phenomena will, he believes, prevent the pulp from undergoing decomposition, even should it die. Teeth are greatly improved in density after fillings of the combined metals have remained in them for a length of time.

*Dr. Davenport*—Has filled proximal cavities by first introducing amalgam, then cutting away the outside surface and filling with gold, but reversing the usual order and working the gold from crown to neck.

*Dr. Dwinelle*—Remarked that lead fillings had been used in sensitive cavities to produce a sedative action on the dentine or pulp. He thinks that amalgam will often save teeth where gold will not, probably because galvanic action causes oxidation and prevents further decay of the tooth-structure, a process which he had frequently referred to as "becoming fossilized." After amalgam fillings have been removed, the dentine is often found to be quite hard, and that too in cases where the tooth-structure was of poor quality before the amalgam was introduced.

*Dr. Clowes*—Asked why it was necessary to mix together these fillings. If amalgam is good, why not use it alone ? He could see no reason for being afraid to employ it.

*Dr. E. H. Raymond* (the Secretary)—Remarked, that when reading the papers under discussion he was struck with the idea that electrical phenomena destroy micro-organisms, and if such is really the case he is decidedly in favor of "giving them more lightning." He spoke of the frequency of using amalgam for repairing cases where gold fillings had broken away, and believed that when carefully done they were quite durable, so much so that some one, in speaking of fillings so repaired, asked: "Did you ever find it necessary to patch the patch?"

*Dr. J. F. P. Hodson*—Believes that combination fillings are very useful in some cases.

*Dr. S. C. G. Watkins,* of Montclair, N. J.—Uses gold and amalgam in combination, and had inserted such fillings at one sitting. Amalgam alone he believes to be too rigid in many cases, and failure is found at the cavity margins. Tin is soft, and when carefully inserted keeps the edges of cavities in good condition and preserves the teeth.

A vote of thanks was given to both Dr. Palmer and Dr. Stockwell for their valuable and interesting papers.

